# AD pathology‐independent effects of astrocyte‐ or microglia‐specific replacement of APOE4 with APOE2

**DOI:** 10.1002/alz.091247

**Published:** 2025-01-03

**Authors:** Georgia L Nolt, Steven M MacLean, Lesley R Golden, Isaiah O Stephens, Gabriela Hernandez, Dahlia Siano, Ellen Woodward, Joshua Moses, Josh M. Morganti, Lance A Johnson

**Affiliations:** ^1^ University of Kentucky, Lexongton, KY USA; ^2^ University of Kentucky, Lexington, KY USA; ^3^ Sanders‐Brown Center on Aging, Lexington, KY USA; ^4^ Univeristy of Kentucky, Lexington, KY USA; ^5^ University of Kentucky College of Medicine, Sanders‐Brown Center on Aging, Lexington, KY USA

## Abstract

**Background:**

Compared with the E3 allele of Apolipoprotein E (APOE), E4 increases late‐onset Alzheimer’s Disease (AD) risk up to 15‐fold, while the E2 allele substantially decreases risk. In the CNS, ApoE is predominantly synthesized by astrocytes and microglia, making these two cell types promising targets for ApoE‐directed therapeutic approaches. Our lab has generated an inducible “switch” mouse model (APOE4s2) in which we can conditionally replace E4 with the protective E2 in a cell‐specific manner. To elucidate potential mechanisms by which astrocytes and microglia expressing E2 or E4may modulate AD risk, we characterized the immuno‐metabolic response of astrocyte‐ and microglia‐specific “switch” mice to a variety of CNS‐related challenges.

**Method:**

Aged APOE4s2 mice were administered tamoxifen to induce an *in vivo* transition from expression of E4 to E2 selectively in astrocytes (*Aldh1l1*‐CreERT2) or microglia (*Tmem119*‐CreERT2). A separate cohort of APOE4s2 mice received LPS to induce an inflammatory response 24 hours prior to tissue collection. Immunohistochemical analysis of gliosis (GFAP, IBA1), cytokine measurements, and targeted metabolomics and lipidomics were performed on brain tissue from these groups. Additionally, a cohort of 6‐week‐old microglia‐APOE4s2 mice were administered lysophosphatidylcholine (LPC) via intracranial injections to promote demyelination. Mice were sacrificed 10 days post‐LPC injection, and myelin content and glial reactivity were quantified via staining of myelin basic protein (MBP), degraded MBP (dMBP), and Luxol Fast Blue (LFB).

**Result:**

As measured by plasma and brain cytokine levels, the inflammatory response was not modified by glial E2 expression in either age or LPS. However, aged E4 mice in which only astrocytes selectively expressed E2 showed decreased microglia reactivity (Iba1 immunoreactivity) compared to mice expressing E4 in all cell types. In contrast, aged mice in which only the microglia express E2 showed increased microglia reactivity relative to E4 expressing controls. Interestingly, a similar increase in Iba1 signal was noted in mice expressing only astrocytic E2 following an acute exposure to LPS. Finally, E2 expressing microglia appear to have higher levels of myelin following LPC injections compared to E4 expressing microglia.

**Conclusion:**

Glial cell‐specific expression of E2 alters microglia reactivity in response to various pathological challenges, including aging, inflammatory stimuli, and demyelination.

